# Birefringence of the Human Cornea: A Review

**DOI:** 10.3390/vision9040090

**Published:** 2025-10-28

**Authors:** Sudi Patel, Larysa Tutchenko, Igor Dmytruk

**Affiliations:** 1 Department of Cataract and Refractive Surgery, University Eye Clinic Svjetlost, Heinzelova 39, 10000 Zagreb, Croatia; 2 Department of Corneal Pathology, Ophthalmic Oncology and Oculoplasty, Kyiv Clinical Ophthalmology Hospital Eye Microsurgery Center, Liubomyra Huzara Ave. 3, 03680 Kyiv, Ukraine; larysatutchenko@gmail.com; 3 Department of Experimental Physics, Faculty of Physics of Taras Shevchenko National University of Kyiv, Hlushkova Ave. 4, 03127 Kyiv, Ukraine; igor_dmytruk@knu.ua

**Keywords:** birefringence, cornea, polarization, refractive index, retinal nerve fiber layer, OCT

## Abstract

Background: This paper aims to provide an overview of corneal birefringence (CB), systematize the knowledge and current understanding of CB, and identify difficulties associated with introducing CB into mainstream clinical practice. Methods: Literature reviews were conducted, seeking articles focused on CB published between the early 19th century and the present time. Secondary-level searches were made examining relevant publications referred to in primary-level publications, ranging back to the early 17th century. The key search words were “corneal birefringence” and “non-invasive measurements”. Results: CB was first recorded by Brewster in 1815. Orthogonally polarized rays travel at different speeds through the cornea, creating a slow axis and a fast axis. The slow axis aligns with the pattern of most corneal stromal collagen fibrils. In vivo, it is oriented along the superior temporal–inferior nasal direction at an angle of about 25° (with an approximate range of −54° to 90°) from the horizontal. CB has been reported to (i) influence the estimation of retinal nerve fiber layer thickness; (ii) be affected by corneal interventions; (iii) be altered in keratoconus; (iv) vary along the depth of the cornea; and (v) be affected by intra-ocular pressure. Conclusions: Under precisely controlled conditions, capturing the CB pattern is the first step in a non-destructive process used to model the ultra-fine structure of the individual cornea, and changes thereof, in vivo.

## 1. Introduction

**Figure 1:**
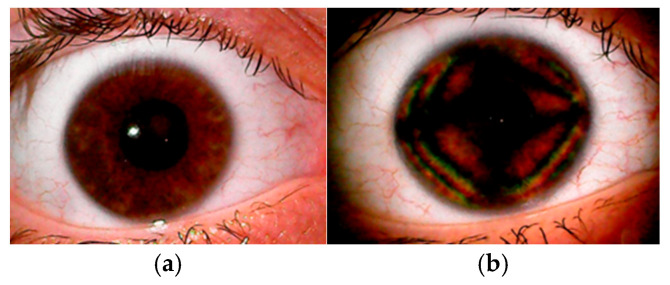
Example of an eye observed using a slit-lamp biomicroscope, at ×10 magnification, (**a**) without polarizers, followed by (**b**) the view with crossed polarizers. On both occasions, the incident beam was in the wide beam setting at 30°, with the subject glancing along the observation beam. For image (**b**) a polarizer with the axis of polarization parallel to the plane of the cornea was mounted in the path of the incident beam and a second polarizer with the axis of polarization perpendicular to the first was placed in the path of the observation beam.

Ocular Coherence Tomography (OCT) and Scanning Laser Polarimetry (SLP) are two game-changing terms that have entered the lexicon of eye care over the last two decades. Fine details of intra-ocular structures are made visible and become amenable to analysis, and the assessment of the retinal nerve fiber layer (RNFL) has become a key feature in the monitoring and overall analysis of the retina. The assessment of the RNFL, like those from the OCT- and SLP-acquired images, is affected by the birefringent properties of the cornea and, to a lesser extent, the crystalline lens [1,2,3,4,5,6,7,8,9,10,11,12,13,14,15,16,17,18,19,20,21,22,23]. The process of estimating the thickness of the RNFL is affected by changes in corneal birefringence following corneal refractive surgery, and this apparent change in the RNFL may be misdiagnosed as resulting from an insidious cause when it is actually an artefact resulting from a change in corneal birefringence [1,3,8,9,10,12,13,14,15,16,17,19,22]. Monitoring corneal birefringence has been proposed as a method for the non-invasive assessment of glucose levels in the anterior chamber [24,25,26,27] and intra-ocular pressure [28,29,30,31,32], though clinically viable instruments that fulfil these roles are not available. Eye care specialists may recognize the term corneal birefringence but may not be familiar with it. So, what is birefringence? Birefringence was first reported to occur in crystals by Bartholin in 1669 [33], and in the ex vivo cornea by Brewster in 1815 [34]; Valentin (in 1861) described seeing colored fringes and a dark cruciform image after placing a human cornea between crossed polarizers [35]. In 1921, Koeppe [36] was the first to use polarized light for the biomicroscopic examination of human corneas in vivo, and Cogan (in 1941) provided more precise descriptions of the dark cross [37]. An example of corneal birefringence consisting of isochromatic zones and dark regions in the shape of a cross is shown in Figure 1.

What causes birefringence? What brings about a change in birefringence? How can corneal birefringence be assessed in a clinical setting? Is there a place for routine assessment of corneal birefringence in a clinical setting? The aim of this review paper was to provide a summary of corneal birefringence with reference to these questions.

## 2. Materials and Methods

This was a narrative review conducted in accordance with established guidelines [38,39]. Primary literature searches were conducted at the British Library and Glasgow Caledonian University Library, in addition to searches on MEDLINE and Google for relevant publications from the early 19th century to the current time. Secondary-level searches were made on relevant publications referenced in primary-level publications stretching back to the early 17th century. The key search words were “cornea birefringence” and “non-invasive measurements”.

### Selection of Material for Review and Data Retrieval

Publications were included for review based on their relevance to the main objective. The included items related to the history and analysis of birefringence, the basic optical requirements for birefringence, properties and classification of corneal birefringence, and the main factors that influence corneal birefringence. Where necessary, numerical information was retrieved for purposes of comparison.

## 3. What Is Birefringence?

Birefringence, or double refraction, occurs when two waves of different states of polarization travel in the same direction through a transmitting medium in which the velocities (and hence, refractive indices) vary depending on the state of polarization. The resulting image is an interference pattern produced in the transmitting medium. A birefringent pattern is observed when the incident light is divided into two sets (ordinary and extraordinary rays) of differing path lengths that are linearly, circularly, or elliptically polarized along mutually orthogonal planes. When these two rays are out of phase by 90°, the light is described as either circularly polarized (when they are of equal amplitude) or elliptically polarized (when the amplitudes are unequal) [40]. The difference in path lengths, termed retardation, depends on the difference in the refractive indices (n_1_ − n_2_) and the thickness (t) of the transmitting medium. The difference (n_1_ − n_2_) also leads to a difference in the phase between the two orthogonally polarized rays of differing path lengths. This phase difference is termed retardance. Hence, the pattern in Figure 1 depends on the polarization, wavelength, and direction of the incident light. Each isochromatic zone is a region in which the retardation, or retardance, is the same. Other than the demonstration of retardation, the cornea has also been credited as having linear, circular, or elliptical polarization properties; characteristics like those observed in uni- and biaxial crystals; and an axis of polarization. These points are explored in more detail under Section 7.1 and Section 8.1.

## 4. What Causes Birefringence?

The root cause of the birefringence could be at a supramolecular level (e.g., in crystalline structure), associated with the structure of the material (e.g., arrangement of fibers), associated with stress (e.g., optical, electrical, magnetic, or mechanical), or a combination of factors. A detailed analysis of the factors and methods suggested for quantifying birefringence is beyond the scope of this review, although it is thoroughly addressed in the standard references [41,42]. The cornea has a layered structure consisting of an outer epithelium, Bowman’s layer, the stroma, Descemet’s layer, and an inner endothelium. The structure of the corneal stroma consists mainly of stacked lamellae in which each single lamella is comprised of a near-parallel arrangement of cylindrical collagen fibers suspended in a medium with a dissimilar refractive index. This fulfils a requirement for birefringence [43,44,45]. The stroma, making up around 90% of the cornea, is generally accepted as the main source of corneal birefringence. Therefore, the corneal birefringence pattern (CBP) can be interpreted as being related to, or representative of, the structure of the stroma.

## 5. What Could Induce a Change in Corneal Birefringence?

Any procedure that alters corneal structure carries the potential to alter the CBP. This includes changes resulting from corneal dystrophies, surgical intervention, or contact-lens wear. For example, thinning of the epithelium [46,47], breaks in Bowman’s layer [46,47,48], compaction and irregular distribution of collagen fibrils [46,48,49], and modulations in the biochemical and biomechanical pathways linked with alterations in collagen cross-linking [50,51] are frequently encountered in the keratoconic cornea. These factors can be expected to distinguish the CBP in keratoconus in comparison with normal cases. Any alterations in these histological characteristics, such as those caused by keratoplasty or contact-lens use, may affect the CBP. The same applies to the normal cornea. The cornea is subjected to ongoing intermittent bursts of stress (e.g., from blinking, eye movements, or rubbing) that could adjust corneal morphology and the CBP on a temporary basis. The cumulative effects of these over several years may account for the reports on changes in the CBP in relation to age [16,52,53] and intra-ocular pressure [28,29,30,31,32]. However, the link between CBP and intra-ocular pressure is questionable [52,53,54].

## 6. How Can the CBP Be Demonstrated and Observed in a Clinical Setting?

The pattern in Figure 1 was produced by placing a polarizer, with the axis of polarization parallel with the plane of the cornea, in the path of the slit-lamp incident light and interposing a second polarizer before the observation beam. Color fringes appear and fade when the second polarizer is rotated. Faint color fringes could be made visible using just one polarizer; however, the pattern is far more intense when two polarizers are used. Specific features of this pattern depend not only on the birefringent properties of the cornea associated with its fine structure, but also on the details of the process used to generate the pattern. Several methods derived from this simple premise have been advanced for the clinical observation of the CBP (see for example Knighton and Huang [55], Misson [56], Lipari et al. [57], and Sobczak et al. [58]). In addition, the SLP [59] and polarization-sensitive OCT [60] can also be used to derive information on corneal birefringence. Full descriptions of these techniques are beyond the scope of this paper. The noted publications should be consulted for further details. However, the key information revealed by the practical applications of these techniques are discussed under Section 7, Section 7.1, Section 8.1, Section 9.1 and Section 9.2.

The fine details of the CBP are systematically dependent upon the mechanism and details of the system used to generate it.

## 7. How Can Corneal Birefringence Be Categorized?

Methods for the more recent clinical assessment of corneal birefringence include SLP, (for example the GDX with variable corneal compensator [5]), polarization-sensitive OCT (for example PS-OCT [61]), modified slit-lamp biomicroscopic techniques [23,54,56,57,58,62,63,64,65,66], and some techniques patented solely for this assessment [23,62,63]. The modus operandi of biomicroscopic techniques is centered mainly on the symmetry of the birefringent pattern [23,54,56,57,58,59,60,61,62,63]. The pattern in Figure 1 features a cross, isochromatic bands arranged in approximate quadrilaterals and dark patches. Every aspect of the pattern is the result of the optical interference of two orthogonally polarized waves propagated with different velocities and phase shifts. After passing through the second polarizer, the waves are transferred to the same polarization plane and interfere. The cross marks the region where only one wave emerges, and hence, optical interference cannot occur. The darker patches may be regions with zero, or very little, interference. The various angles, displacement of the cross, shape and orientation of the cross, asymmetry, and dimensions of the isochromatic quasi-quadrilaterals have been used to categorize and quantify corneal birefringence [23,42,43,54,57,58,63]. Irregularities of the cross and other features in Figure 1 could be attributed to circular or elliptical polarization in conjunction with linear polarization. SLP- and PS-OCT-based systems for assessing CB rely on precision control of the polarization of light entering the eye, fine tuning the analysis of light back-scattered out of the eye, with algorithms suitably adapted to compensate for birefringent activity occurring in the macula, and the wavelength of the incident light (≈780 nm for SLP, ≈840 nm for PS-OCT). The technical details of these systems are fully described in other publications [59,60]. These systems generate maps that display CB activity across multiple locations over the cornea, values which can subsequently be compounded into single figures. This is further explored in Section 8.1. The nuances and fine features of the observed pattern depend not only on the way the pattern is generated but also on how the observed image is processed and the limitations of the applied algorithms.

### 7.1. Does the Cornea Have Characteristics of a Uni- or Biaxial Crystal?

A uniaxial crystal is an anisotropic medium with an optic axis where incident rays parallel to this axis exhibit no birefringence [41,42]. The velocity and the refractive index along this direction are not related to the polarization of the propagated rays [67]. Uniaxial crystals have two refractive indices along orthogonal axes. Biaxial crystals have, as stated by Stoiber and Morse, *two optic axes where rays travel at the same velocity. In every other wave propagation direction there are two linearly polarized components at right angles to each other and to the propagation direction; these have unequal velocities* [68]. Therefore, CB could be categorized as a uni- or biaxial crystal. According to Stanworth and Naylor, the cornea is analogous to a bent uniaxial crystal [28,29], though more recent studies suggest the cornea is more akin to a biaxial crystal [69,70,71,72,73,74] and the birefringence is thickness-dependent [17,54,75,76]. The structure of the cornea in the region under examination and the treatment meted out to the cornea will determine if it has uni- or biaxial properties. An individual cornea may be erroneously identified as having properties of a uniaxial crystal when the angle between the two optic axes is not detected. Categorizing the cornea as uni- or biaxial is of limited value to the clinician interested in the structure of the cornea.

## 8. How Can Corneal Birefringence Be Quantified for Analysis and Statistical Purposes?

### 8.1. Orientation of the Slow Axis, Estimating Corneal Retardation and Differences in the Refractive Index (n_1_ − n_2_)

All aspects of the CBP are amenable to statistical analysis. Some of the published material is laden with jargon and difficult to interpret. However, monitoring the orientation of the slow axis of the cornea is a relatively simple way of interpreting the CBP by reducing the complex pattern to a single number. But what does the “slow axis” represent? A birefringent medium features slow and fast axes because orthogonally polarized rays travel at different velocities through it. The retardation of light is greatest along the length of a collagen fibril [38,39]. Therefore, n_1_ − n_2_ is the difference in the refractive indices along the length and across the collagen fibril, and the orientation of the slow axis of the cornea indicates the main direction of stromal collagen alignment.

The orientations of the slow and fast axes can be identified by placing the birefringent medium between two crossed polarizers (at 90° and 180°) and passing light through the three-part combination. The light emerging from the second polarizer is gradually extinguished when the medium is rotated about an axis parallel with the direction of the incident light. The transmitted light is extinguished when the retardation equals half the wavelength of the incident ray (λ/2) or multiples thereof. At this point the orientation of the birefringent material axis is at 45° relative to the mutually perpendicular slow and fast axes. It is worth considering the clinical procedure presented by Knighton and Huang [55] to determine the angle of the slow axis of the cornea in vivo, because it was based on the same principles. This involved observing the fourth Purkinje image during slit-lamp microscopy. The polarizer before the light incident on the cornea is kept perpendicular to the polarizer placed along the observation beam. The instrument is engineered to keep the two polarizers mutually perpendicular while the first polarizer is rotated. The fourth Purkinje image is observed, and the angle of the slow axis is determined by rotating the first polarizer until the fourth Purkinje image is no longer visible. At this point, the axis of the first polarizer is either aligned with, or perpendicular to, the slow axis of the cornea. The polarizers are then rotated by 45°, making the fourth Purkinje image visible and a commercially available, pre-calibrated, variable retarder is placed before the observation beam aligned with the slow axis and the retardation is adjusted until the fourth Purkinje image is extinguished. If the visibility of the fourth Purkinje image persists, then the variable retarder is turned through 90° and the process is repeated, because the retarder had mistakenly been placed along the fast axis. The reading on the variable retarder is an estimate of the corneal retardation for the region of the cornea under observation. The exact value of the retardation could vary over the surface of the cornea under observation. The reading on the retarder could be used to estimate the difference in refractive index (n_1_ − n_2_) if the actual thickness perpendicular to the outer surface of the cornea is known. Based on a variety of assumptions, including an overall average corneal refractive index of 1.377 and an apparent corneal thickness, Misson predicted this difference was ≈ 0.00159 [77]. This was a step towards modeling corneal structure, i.e., advancing a model of corneal structure to account for the observation. However, the literature on corneal birefringence still raises questions concerning the source, classification, and interpretation of the observed pattern.

A separate optical retarder can be included as part of the optical set up for detecting corneal birefringence. Commercially available pre-calibrated optical retarders have been used to either neutralize or change the appearance of corneal birefringence to a pre-set pattern. So, the angle of the slow axis (some authors refer to this as the polarization axis, [4,14,52,53,77]) of the corneal birefringence pattern can be estimated by adjusting the separate optical retarder to a predetermined level. Some of the key publications on estimating the orientation of the slow axis of the human cornea are noted in Table 1. The main direction of the stromal collagen fibrils is commensurate with the slow axis, and so the slow-axis values in Table 1 are indicative of corneal structure where collagen fibers are orientated mainly along the superior temporal–inferior nasal direction. The angle can be interpreted as a single-figure description of corneal structure. There is a similarity in the mean reported values, but the wide range of values implies there are substantial differences between corneas, or the computations are prone to systematic errors, or the reliability of the procedures is questionable. Nevertheless, a change in this angle in an individual case, e.g., following a corneal procedure, could be interpreted as an alteration in the fine structure of the tissue. This is further explored in Section 9.1 and Section 9.2. Changes in the orientation of the slow axis correlate with the depth of ablation following LASIK [17]. This suggests the birefringent properties vary along the depth of the cornea. The disruption resulting from exposure to the photoablating beam, coupled with the subsequent healing response, cannot be dismissed as the prime cause of any shift in the orientation. The apparent change in the birefringence may be an outcome dependent on the applied correction. However, if there is a genuine depth-dependency, then the birefringence in a dystrophic cornea should differ from the norm.

### 8.2. Matrix Analysis of Birefringent Patterns

The passage of polarized light through a medium can be described using either the Jones or the Mueller matrix analytical procedures. These have been used to further explore the properties of corneal birefringence, and the information generated by their usage has resulted in the construction of models of the cornea [27,58,64]. The specific information revealed by these analyses can be difficult to interpret without an in-depth knowledge and understanding of matrix analysis and applications thereof. These matrices are mathematical tools for image analysis, and they have their limitations. The Mueller calculus is useful for describing depolarized or partially polarized light, while the Jones calculus can describe fully polarized light. Details of corneal birefringence revealed by these tools will depend on the specific characteristics of the polarized light directed into, and passing out of, the cornea. Thus, according to Shurcliff, the Mueller calculus is useful for understanding the particular characteristics of the cornea that lead to polarization activity, characteristics that the Jones calculus does not provide [79]. On the other hand, the Jones matrix will reveal information about properties of the cornea that the Mueller calculus cannot [79].

## 9. Can the CBP Be Used to Model Corneal Structure?

Prior to proceeding further, it is important to note that Pierscionek and Weale found there was almost complete neutralization of the corneal cross when a gonioscopy lens was applied to a small number of normal and glaucomatous eyes [65]. They concluded the reflection at the external corneal surface was the main contributor to the observed pattern. This raises the question of whether the pattern in Figure 1 is primarily influenced by reflections at the external corneal surface or by corneal structure. If the pattern in Figure 1 is the result of reflectance at the external corneal surface, then

(i) Fresnel equations should predict the intensity distribution of the polarized light reflected off the ocular surface [80];
(ii) Characteristics of the dark cross over the surface of a powered contact lens placed on the eye should differ in comparison with the dark cross over the ocular surface;
(iii) The characteristics of the cross should be markedly different in cases of ectasia;
(iv) The appearance of the cross should change in response to changes in the characteristics of the precorneal tear film during the interblink interval.

**Figure 2:**
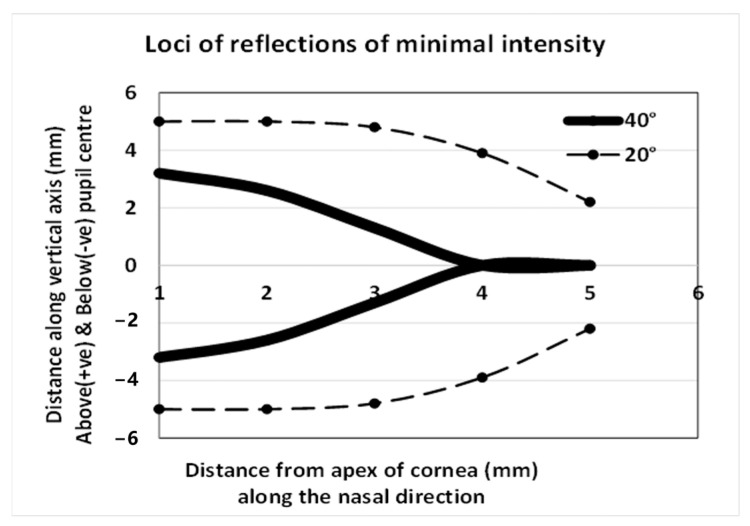
Estimated regions where the specular reflections have minimal intensity when the ocular surface is illuminated with light linearly polarized in a direction parallel to the plane of reflection, from the temporal side, and at angles of 20° or 40°. These regions gradually collapse towards the horizontal axis along the nasal direction from the pupil center.

Interestingly, the Fresnel equations have been used for the non-invasive estimation of the refractive index of the corneal epithelium. The values ranged from 1.410 to 1.416 [81,82]. The outermost surface of the cornea is the lipid layer of the precorneal tear film, and the refractive index of the lipid is about 1.482 [83]. Thus, it is reasonable to adopt this value when estimating the intensity of light reflected by the ocular surface. Figure 2 shows the regions where the intensities of reflections are minimal when a 10 mm wide beam of linearly polarized light (polarized in a direction parallel to the plane of reflection) is incident upon the cornea from the temporal side at an angle of 20° or 40°. The form of the dark region depends on the angle of illumination and is located on the nasal side of the pupil. The calculations underlying Figure 2 are shown in the appendix [Appendix A]. The predictions do not resemble Figure 1. Reflections from the ocular surface are discounted, as the structure chiefly determines the pattern observed in Figure 1. The pattern in Figure 1 is remarkably stable during the inter-blink interval, and it is on par with the images obtained from ex vivo samples by light transmission [70,84,85,86]. Thus, the internal architecture of the cornea is the chief determinant of this image. The CBP should not occur when incident rays strike the cornea perpendicular to the surface, as the instances of birefringence formed within the lamellae are expected to cancel each other out when the stromal fibers are uniformly distributed within each lamella and the lamellae are stacked randomly on top of each other [87]. The fact that corneal birefringence is observed in vivo indicates there is some order in the arrangement of the stromal lamellae, unless it is an artefact.

### 9.1. Modelling the Architecture of the Corneal Stroma

The values for the slow axis of the cornea in Table 1 indicate the alignment of stromal collagen fibers. The procedure described by Knighton and Huang utilizes information obtained from a small paracentral region along the horizontal axis of the cornea [55]. Thus, the value for the slow axis is an indicator of the orientation of stromal fibers in this small paracentral region. Corneal birefringence determinations acquired by SLP and PS-OCT cover the central and paracentral regions. Therefore, the information obtained by SLP and PS-OCT could be used to build up more comprehensive models of stromal structure covering the whole cornea. Bueno et al. described an experimental combined aberrometer and polariscope arrangement used to investigate the birefringence within the pupil [88]. In a small number of eyes (four normal, four post-LASIK), they found the slow axis was directed mainly along the superior temporal—inferior nasal direction, with variations over the pupil area. In this small group, there was a shift in the orientation of stromal fibers over the central pupillary zone. On an individual case-by-case basis, there was a centrifugal change in the retardation from the center of the cornea [89], and it appears this is linked to pupil size [90]. No major large-scale studies have been reported either supporting or refuting these claims. This suggests the stromal structure varies topographically from the corneal apex to the limbus.

The demonstration in Figure 1 requires light to be reflected by the iris. If the information revealed is affected by the nature of this reflection, then it may be the case that the pattern will change with dilation, be pupil-related, and depend on the quality of the iris surface. A search to find definitive studies linking the quality of the CBP with the texture or appearance of the iris did not meet with success. However, a study based on six subjects reported pharmacologically induced constriction, or dilation, of the pupil-adjusted corneal birefringent characteristics [91]. The implication is that the shift in pupil dynamics induces stress in the cornea or adjusts the fine structure of the cornea, or that the pattern is linked with light scattered/diffracted by the surface of the iris or the optical properties of the other intraocular structures.

The CBP is the result of a myriad of interactions between the light scattered within the ocular media and then reflected out of the eye. Therefore, it is reasonable to expect the pattern to differ in the pseudophakic eye where the surface of the intra-ocular lens is smoother, flatter, and more reflective, compared with the natural crystalline lens.

Misson used circularly polarized light to investigate corneal structure in fully dilated eyes. The CBP observed over the cornea consisted of two quasi-circular dark patches aligned along the superior temporal–inferior nasal direction, and fine lines [56,62]. The orientation of a line drawn through the centers of the dark patches is comparable with the slow-axis values noted in Table 1. This orientation can be interpreted as representing the average direction of the stromal fibers. The fine lines were recognized as representing bundles of stromal fibers. Furthermore, the distribution of these fine lines was typically elliptical, or pear shaped. These fine lines mirror the fine architecture of the stromal fibers, and there were no obvious differences in the patterns obtained from phakic and pseudophakic eyes.

Moreover, the details of the patterns were not affected by the characteristics of the rays passing out through the pupil after traversing and reflecting off other intra-ocular structures. Under controlled conditions, the fine details of corneal birefringence patterns can be mapped out by suitably filtering and analyzing the captured images [20,32,56,62,72]. The validity of the map is reliant upon the reliability of the corneal birefringence image, the robustness of the algorithms used, and the cumulative systematic error resulting from the various assumptions made during the analytical process. Nevertheless, Misson’s results stem from the presumption that the stromal structure has a common feature in all corneas [53,59]. The finer details, like fingerprints, are unique to each cornea.

### 9.2. Is the CBP Representative of Corneal Thickness, Distribution, or Structure?

Centofanti et al. found that a change in the angle of the slow axis correlated with the depth of ablation following LASIK [17]. This suggests that the extent of the intervention affects the changes in the orientation of the stromal fibers or that the orientation varied along the depth of the cornea. If there is a genuine depth dependency, then the birefringence in unusually thick or thin corneas should differ from the norm. The CBP is altered in keratoconus [20,56,76] and after various forms of corneal surgery [1,3,8,9,10,12,13,14,15,16,17,18,19,22,85]. Differences in the CBP may depend on the overall structure or be related to variations in the distribution of corneal thickness [17,54,75,76]. If corneal thickness is the main contributor to the CBP, then the pattern should change as an increase in thickness distribution is induced by increasing corneal hydration (e.g., by bathing the cornea with distilled water) or in Fuchs’ dystrophy. A search of the literature failed to identify any publication in which changes in CBP were linked to corneal hydration. On the other hand, if the CBP is a direct consequence of corneal thickness distribution, then the physical properties of the pattern should invariably correspond with a map of corneal thickness distribution. Any disconnect between the two would, by process of elimination, be related to other factors or details pertaining to corneal structure.

The models of corneal structure constructed from the CBP all describe patterns of stromal fiber distribution. These models include descriptions of fibers arranged along circumferential orientations [85] and passing from the apex towards the limbus along radial lines [71]; stromal lamellae oriented mainly along two orthogonal axes, with the remainder arranged randomly [76]; and fibers arranged predominantly along elliptical lines along the direction of the slow axis, with the remainder organized in discrete fingerprint-like patterns [55,56,87]. The models describe fibrillar distribution in the *x*–*y* plane over the surface of the cornea, not along the depth of the cornea. The patterns of corneal structure derived from the CBP do not appear to be influenced by corneal thickness distribution.

## 10. Conclusions

The major clinical entities associated with corneal birefringence are listed in Table 2. With new techniques for modifying corneal optics, artificial implants, and advancing xeno-transplantation [92], there is a need for quick clinical procedures for modeling and monitoring corneal structure. The validity and reliability of emerging technologies must be established, and the limits of agreement between techniques should be determined. The information that the CBP delivers remains unused by many clinicians. However, the non-invasive CBP images are easy to demonstrate and capture for analytical purposes. The determination of the CBP enables the creation of a profile, equivalent to a fingerprint, of individual corneal structure, one beyond the limits of OCT. This is analogous to working out the DNA profile of an individual. Alternatively, a library of hypothetical CBPs could be assembled based on a range of distribution patterns of stromal collagen fibrils. Matching the hypothetical CBP with the actual CBP could be a step towards categorizing the specific distribution of stromal collagen fibrils of the individual. The contributions, if any, of other features of the cornea on the CBP have yet to be fully understood. The epithelium is optically distinct from the stroma and makes its own contribution to the overall refractive properties of the eye. Thus, there is the possibility for changes within the epithelium to affect the overall corneal birefringence pattern. For now, models of stromal architecture can be formulated to account for a CBP and changes thereof.

## Figures and Tables

**Table 1:** Some of the key values for the angle of the slow axis of the human cornea published since 2000.

Authors	Year	Technique	Angle of Slow (Polarization) Axis (°)
Greenfield et al. [4]	2000	Slit lamp/4th Purkinje Image	Mostly 10 to 20 (−54 to 90), *n* = 112
Greenfield and Knighton [77]	2001	Slit lamp/4th Purkinje Image	24 ± 18 (−13 to 67), *n* = 71. One year later, 21 ± 15 (−14 to 59)
Weinreb et al. [52]	2002	GDx™ with NFL analyser	24.5 ± 17.4 (−13 to 73), R = 40 ± 15.7 nm (7–90), *n* = 55 *
Knighton and Huang [55]	2002	Slit lamp/4th Purkinje Image	(10 to 40), R = (40–140) nm, *n* = 73
Angeles et al. [14]	2004	GDx-VCC™	31.5 (CI 27.7 to 37.3), R = 41.6 nm (CI 36.6 to 46.5), *n* = 37
Knighton et al. [59]	2008	GDx-VCC™	10 to 40, *n* = 21
Irsch and Shah [78]	2012	GDx-VCC™	23 ± 17 (−11 to 71), R = 39 nm ± 16 (10 to 77) ^1^ 10 ± 13 (−6 to 51), R = 39 nm ± 17 (7 to 78) ^2^

Mean and ± s.d. values are noted where readily available, *n* = number of eyes, R = retardation (nm). Figures in parentheses are ranges. A positive value indicates the direction is along the superior temporal–inferior nasal direction, a negative value indicates the direction is along the lower temporal–upper nasal direction, *n* = number of eyes, R = retardation (nm). * = significant correlation between angle of slow (polarization) axis and/or retardation with age in glaucoma. ^1^ In children aged 3–18 years. ^2^ In adults aged 18+ years. CI = range of 95% confidence interval, NFL = nerve fiber layer, GDx = scanning laser polarimeter, GDx-VCC = scanning laser polarimeter with a variable corneal compensator, GDx and GDx-VCC = estimates of the polarization values for wavelength (λ) = 780 nm. Other values are measurements for visible wavelengths (λ ≈ 585 nm). Some authors refer to the slow axis as the polarization axis.

**Table 2:** Summary of the main clinical features associated with corneal birefringence patterns.

Characteristic Feature	Supportive Publications
Corneal architecture influences the CBP	[45,55,56,62,70,88,91]
The cornea is akin to a biaxial crystal	[69,70,71,72,73,74]
CBP may not be affected by intra-ocular pressure	[52,53,54]
CBP can be used to model the structure of the central cornea in vivo	[56,62,88,89]
CBP is altered in the abnormal corneas	[1,3,8,9,10,12,13,14,15,16,17,18,19,20,22,56,75,90]
CBP is affected by corneal refractive procedures	[1,3,8,9,10,12,13,14,15,16,17,18,19,22,89]

CBP = Corneal birefringence pattern.

**Figure A1:**
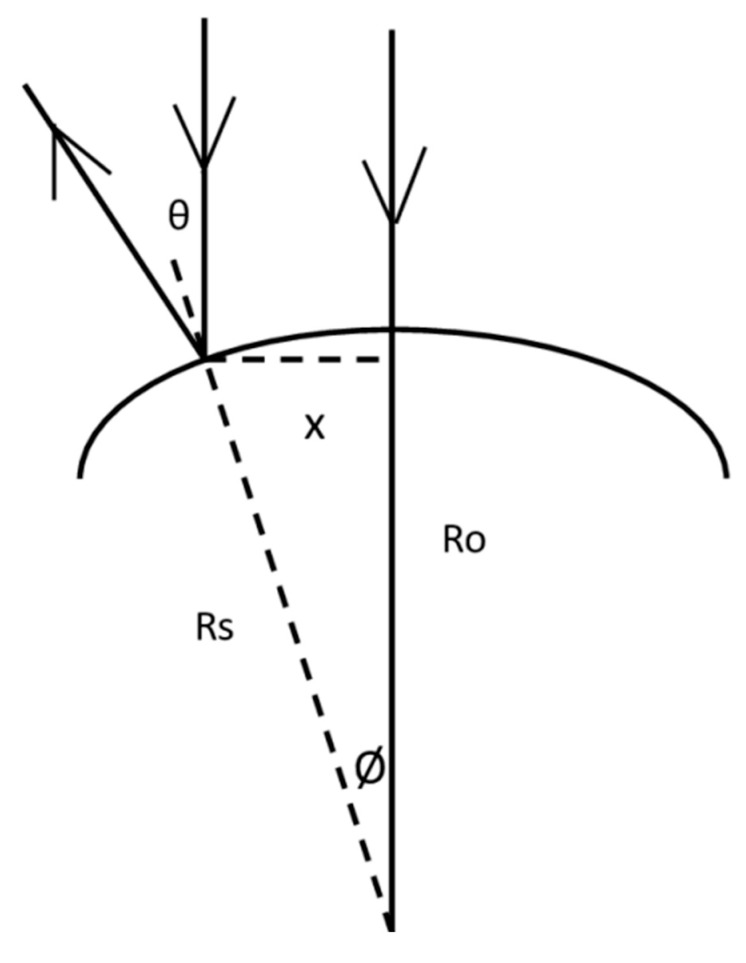
An aspheric corneal surface, rotationally symmetrical about the axis, with the normal beam passing through the apex, and a parallel beam incident along the axis normal to the apex.

**Figure A2:**
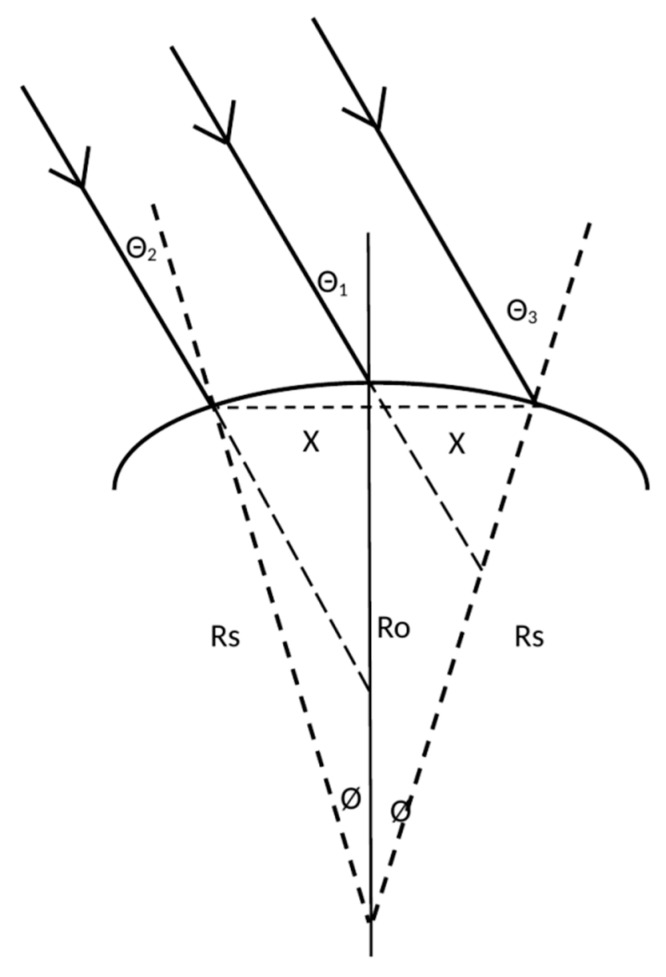
A parallel beam incident at an angle of Θ_1_ to the normal, passing through the apex of the cornea.

**Figure A3:**
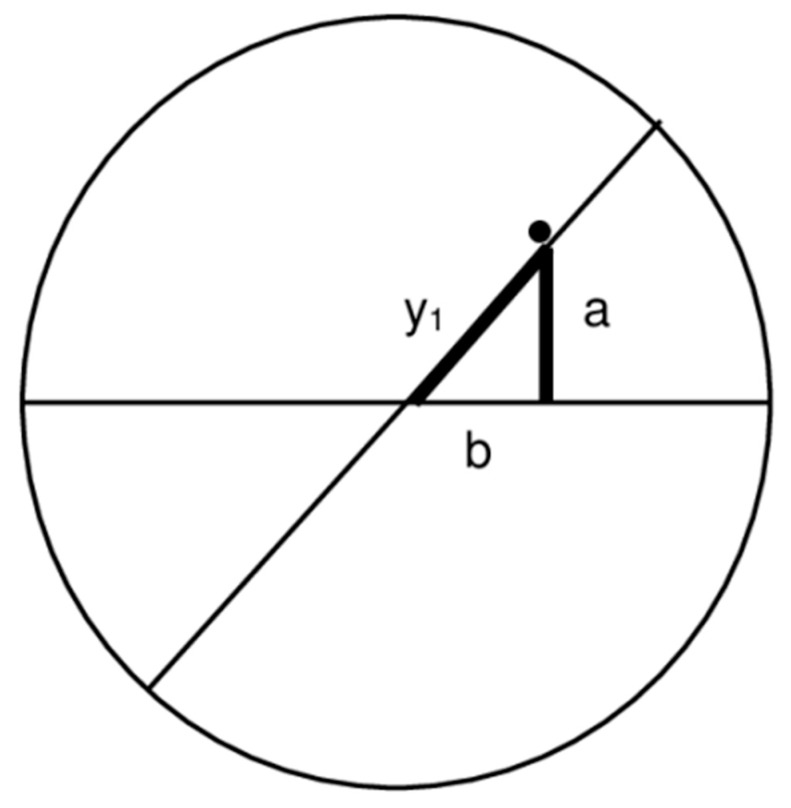
A wide parallel beam incident at an angle of Θ_1_ to the normal at the apex of the cornea. The first ray strikes the apex of the cornea. A second ray strikes at • distant y_1_ from the apex.

## Data Availability

This study did not include collection of data from subjects, patients, or laboratory bench tests.

## Abbreviations

The following abbreviations are used in this manuscript:

CB	Corneal birefringence
CBP	Corneal birefringence pattern
SLP	Scanning laser polarimetry or Scanning laser polarimeter
OCT	Ocular coherence tomography or Ocular coherence tomographer
RNFL	Retinal nerve fiber layer
LASIK	Laser-assisted in situ keratomileusis
n_1_ − n_2_	Difference in the refractive indices
t	Corneal thickness
Θ	Angle of incidence
Ψ	Angle of refraction
Ro	Radius at the apex of the cornea
Rs	Sagittal radius of the cornea at distance x from the apex

## Appendix A

Fresnel equations estimate the relative intensity of light reflected off a dielectric surface [80]. The relative intensity of the reflection for incident light linearly polarized in a direction parallel to the plane of reflection is

R_‖_ = tan^2^ (Θ − ψ)/tan^2^ (Θ + ψ)

And the relative intensity of the reflection for incident light linearly polarized in a direction perpendicular to the plane of reflection is

R_⟂_ = sin^2^ (Θ − ψ)/sin^2^ (Θ + ψ)

In both equations, Θ = angle of incidence, ψ = angle of refraction.

Figure A1 shows, for an aspheric surface with rotational symmetry about the axis, with a normal beam passing through the apex and a parallel beam incident normal to the apex, Θ = Ø and sin Ø = x/Rs.

According to Baker [93], Rs = √[(Ro)^2^ + (1 − p)x^2^], where Ro = radius at the apex of the cornea, x = distance from apex, and p = shape factor describing the asphericity of the surface.

∴ Θ = arcsin {x/√[(Ro)^2^ + (1 − p)x^2^]}.

For a parallel beam incident at an angle of Θ_1_ to the apex (see Figure A2),

Θ_2_ = Ø − Θ_1_ & Θ_3_ = Ø + Θ_1_

∴ Θ_2_ = arcsin {x/√[(Ro)^2^ − (1 − p)x^2^]} − Θ_1_
(A1)

& Θ_3_ = arcsin {x/√[(Ro)^2^ + (1 − p)x^2^]} + Θ_1_
(A2)

For a wide parallel beam incident at an angle of Θ_1_ to the apex (see Figure A3), the first ray strikes the apex of the surface. A second ray strikes at • distant y_1_ from the apex,

y_1_ = √(a^2^) + (b^2^).

For an aspheric surface with rotational symmetry about the axis, the beam passing through the apex, x is replaced by y_1_ in Equations (A1) and (A2), along the hatched axis, where Rs now = √[(Ro)^2^ + (1 − p)y_1_^2^]

Θ_2_ now = arcsin {[√(a^2^) + (b^2^)]/Rs} − Θ_1_ and Θ_3_ now = arcsin {[√(a^2^) + (b^2^)]/Rs} + Θ_1_

y_1_ = √(a^2^) +(b^2^).

## References

[1] Choplin N.T., Schallhorn S.C. (1999). The effect of excimer laser photorefractive keratectomy for myopia on nerve fiber layer thickness measurements as determined by scanning laser polarimetry. Ophthalmology.

[2] Collur S., Carroll A.M., Cameron B.D. (2000). Human lens effect on in vivo scanning laser polarimetric measurements of retinal nerve fiber layer thickness. Ophthalmic Surg. Lasers.

[3] Gürses-Ozden R., Pons M.E., Barbieri C., Ishikawa H., Buxton D.F., Liebmann J.M., Ritch R. (2000). Scanning laser polarimetry measurements after laser-assisted in situ keratomileusis. Am. J. Ophthalmol..

[4] Greenfield D.S., Knighton R.W., Huang X.R. (2000). Effect of corneal polarization axis on assessment of retinal nerve fiber layer thickness by scanning laser polarimetry. Am. J. Ophthalmol..

[5] Kogure S., Chiba T., Kinoshita T., Kowa H., Tsukahara S. (2000). Effects of artefacts on scanning laser polarimetry of retinal nerve fibre layer thickness measurement. Br. J. Ophthalmol..

[6] Knighton R.W., Huang X.R., Greenfield D.S. (2002). Analytical model of scanning laser polarimetry for retinal nerve fiber layer assessment. Investig. Ophthalmol. Vis. Sci..

[7] Garway-Heath D.F., Greaney M.J., Caprioli J. (2002). Correction for the erroneous compensation of anterior segment birefringence with the scanning laser polarimeter for glaucoma diagnosis. Investig. Ophthalmol. Vis. Sci..

[8] Roberts T.V., Lawless M.A., Rogers C.M., Sutton G.L., Domniz Y. (2002). The effect of laser-assisted in situ keratomileusis on retinal nerve fiber layer measurements obtained with scanning laser polarimetry. J. Glaucoma.

[9] Holló G., Nagy Z.Z., Vargha P., Süveges I. (2002). Influence of post-LASIK corneal healing on scanning laser polarimetric measurement of the retinal nerve fibre layer thickness. Br. J. Ophthalmol..

[10] Nevyas J.Y., Nevyas H.J., Nevyas-Wallace A. (2002). Change in retinal nerve fiber layer thickness after laser in situ keratomileusis. J. Cataract. Refract. Surg..

[11] Choplin N.T., Zhou Q., Knighton R.W. (2003). Effect of individualized compensation for anterior segment birefringence on retinal nerve fiber layer assessments as determined by scanning laser polarimetry. Ophthalmology.

[12] Holló G., Katsanos A., Kóthy P., Kerek A., Süveges I. (2003). Influence of LASIK on scanning laser polarimetric measurement of the retinal nerve fibre layer with fixed angle and customised corneal polarization compensation. Br. J. Ophthalmol..

[13] McCarty T.M., Hardten D.R., Anderson N.J., Rosheim K., Samuelson T.W. (2003). Evaluation of neuroprotective qualities of brimonidine during LASIK. Ophthalmology.

[14] Angeles R., Abunto T., Bowd C., Zangwill L.M., Schanzlin D.J., Weinreb R.N. (2004). Corneal changes after laser in situ keratomileusis: Measurement of corneal polarization magnitude and axis. Am. J. Ophthalmol..

[15] Choplin N.T., Schallhorn S.C., Sinai M., Tanzer D., Tidwell J.L., Zhou Q. (2005). Retinal nerve fiber layer measurements do not change after LASIK for high myopia as measured by scanning laser polarimetry with custom compensation. Ophthalmology.

[16] Bagga H., Greenfield D.S., Feuer W.J. (2005). Quantitative assessment of atypical birefringence images using scanning laser polarimetry with variable corneal compensation. Am. J. Ophthalmol..

[17] Centofanti M., Oddone F., Parravano M., Gualdi L., Bucci M.G., Manni G. (2005). Corneal birefringence changes after laser assisted in situ keratomileusis and their influence on retinal nerve fibre layer thickness measurement by means of scanning laser polarimetry. Br. J. Ophthalmol..

[18] Tóth M., Holló G. (2005). Enhanced corneal compensation for scanning laser polarimetry on eyes with atypical polarisation pattern. Br. J. Ophthalmol..

[19] Shoji T., Takahashi H., Park M., Okazaki K., Tanito M., Chihara E. (2007). Prospective evaluation of factors associated with post-LASIK corneal birefringence with scanning laser polarimetry. J. Glaucoma.

[20] Götzinger E., Pircher M., Dejaco-Ruhswurm I., Kaminski S., Skorpik C., Hitzenberger C.K. (2007). Imaging of birefringent properties of keratoconus corneas by polarization-sensitive optical coherence tomography. Investig. Ophthalmol. Vis. Sci..

[21] Kogure S., Kohwa H., Tsukahara S. (2008). Effect of uncompensated corneal polarization on the detection of localized retinal nerve fiber layer defects. Ophthalmic Res..

[22] Aristeidou A.P., Labiris G., Paschalis E.I., Foudoulakis N.C., Koukoula S.C., Kozobolis V.P. (2010). Evaluation of the retinal nerve fiber layer measurements, after photorefractive keratectomy and laser in situ keratomileusis, using scanning laser polarimetry (GDX VCC). Graefes Arch. Clin. Exp. Ophthalmol..

[23] Iovieno A., Yeung S.N., Nahum Y., Teichman J., Lipari E., Busin M., Fontana L. (2016). Polarimetric Interferometry for Assessment of Corneal Stromal Lamellae Orientation. Cornea.

[24] Baba J.S., Cameron B.D., Theru S., Coté G.L. (2002). Effect of temperature, pH, and corneal birefringence on polarimetric glucose monitoring in the eye. J. Biomed. Opt..

[25] Malik B.H., Coté G.L. (2010). Modeling the corneal birefringence of the eye toward the development of a polarimetric glucose sensor. J. Biomed. Opt..

[26] Pirnstill C.W., Malik B.H., Gresham V.C., Coté G.L. (2012). In vivo glucose monitoring using dual-wavelength polarimetry to overcome corneal birefringence in the presence of motion. Diabetes Technol. Ther..

[27] Westphal P., Kaltenbach J.M., Wicker K. (2016). Corneal birefringence measured by spectrally resolved Mueller matrix ellipsometry and implications for non-invasive glucose monitoring. Biomed. Opt. Express.

[28] Stanworth A., Naylor E.J. (1953). Polarized light studies of the cornea I. The isolated cornea. J. Exp. Biol..

[29] Stanworth A. (1953). Polarized light studies of the cornea II. The effect of intra-ocular pressure. J. Exp. Biol..

[30] Yamanari M., Nagase S., Fukuda S., Ishii K., Tanaka R., Yasui T., Oshika T., Miura M., Yasuno Y. (2014). Scleral birefringence as measured by polarization-sensitive optical coherence tomography and ocular biometric parameters of human eyes in vivo. Biomed. Opt. Express.

[31] Richhariya A., Verma Y., Rao D.K., Roberts C.J., Mahmoud A.M., Sangwan V.S., Punjabi S., Gupta P.K. (2014). Effect of Intraocular Pressure and Anisotropy on the Optical Properties of the Cornea: A Study Using Polarization Sensitive Optical Coherence Tomography. Asia-Pacific J. Ophthalmol..

[32] Ke L., Zhang L., Zhang N., Wu Q.Y.S., Leong H.S., Abdelaziem A., Mehta J.S., Liu Y.-C. (2022). Corneal elastic property investigated by terahertz technology. Sci. Rep..

[33] Bartholin E., Archibald T. (1991). Experiments with the Birefringent Icelandic Crystal, Which Led to the Discovery of a Wonderful and Extraordinary Refraction.

[34] Brewster D. (1815). Experiments on the de-polarization of light as exhibited by various mineral, animal and vegetable bodies with a reference to the general principles of polarization. Phil Trans. R. Soc. Lond..

[35] Valentin G. (1861). Die Untersuchung der Pflanzen- und der Thiergewebe in Polarizirtem Lichte.

[36] Koeppe L. (1921). Die Ultra und Polarizationsmikroskopische Erforschung des Lebenden Auges und Ihre Ergebnisse.

[37] Cogan D.G. (1941). Some Ocular Phenomena Produced with Polarized Light. Arch. Ophthal..

[38] Murphy C.M. (2012). Writing an Effective Review Article. J. Med. Toxicol..

[39] Sukhera J. (2022). Narrative Reviews: Flexible, Rigorous, and Practical. J. Grad. Med. Educ..

[40] Douplik A., Saiko I.G., Schelkanova I., Tuchin V.V., Jelínková H. (2013). The response of tissue to laser light. Lasers for Medical Applications: Diagnostics, Therapy and Surgery.

[41] Longhurst R.S., Longhurst R.S. (1967). The propagation of light in crystals. Geometrical and Physical Optics.

[42] Born M., Wolf E., Born M., Wolf E. (1980). Interference and diffraction with partially coherent light. Principles of Optics.

[43] Daxer A., Fratzl P. (1997). Collagen fibril orientation in the human corneal stroma and its implication in keratoconus. Investig. Ophthalmol. Vis. Sci..

[44] Meek K.M., Quantock A.J. (2001). The use of x-ray scattering techniques to determine corneal ultrastructure. Prog. Retin. Eye Res..

[45] Bour L.J., Charman W.N. (1991). Polarized light and the eye. Visual Optics and Instrumentation.

[46] Fernandes B.F., Logan P., Zajdenweber M.E., Santos L.N., Cheema D.P., Burnier M.N. (2008). Histopathological study of 49 cases of keratoconus. Pathology.

[47] Sykakis E., Carley F., Irion L., Denton J., Hillarby M.C. (2012). An in depth analysis of histopathological characteristics found in keratoconus. Pathology.

[48] Fournié P., Touboul D., Arné J.L., Colin J., Malecaze F. (2013). Kératocône [Keratoconus]. J. Fr. Ophtalmol..

[49] Meek K.M., Tuft S.J., Huang Y., Gill P.S., Hayes S., Newton R.H., Bron A.J. (2005). Changes in collagen orientation and distribution in keratoconus corneas. Investig. Ophthalmol. Vis. Sci..

[50] Shetty R., D’Souza S., Khamar P., Ghosh A., Nuijts R.M.M.A., Sethu S. (2020). Biochemical Markers and Alterations in Keratoconus. Asia Pac. J. Ophthalmol..

[51] Singh R.B., Koh S., Sharma N., Woreta F.A., Hafezi F., Dua H.S., Jhanji V. (2024). Keratoconus. Nat. Rev. Dis. Primers.

[52] Weinreb R.N., Bowd C., Greenfield D.S., Zangwill L.M. (2002). Measurement of the magnitude and axis of corneal polarization with scanning laser polarimetry. Arch. Ophthalmol..

[53] Mai T.A., Lemij H.G. (2008). Longitudinal measurement variability of corneal birefringence and retinal nerve fiber layer thickness in scanning laser polarimetry with variable corneal compensation. Arch. Ophthalmol..

[54] Sobczak M., Asejczyk M., Kalinowski K., Pierścionek B. (2021). Comparative analysis of the corneal birefringence pattern in healthy children and adults. Ophthalmic Physiol. Opt..

[55] Knighton R.W., Huang X.R. (2002). Linear birefringence of the central human cornea. Investig. Ophthalmol. Vis. Sci..

[56] Misson G.P. (2007). Circular polarization biomicroscopy: A method for determining human corneal stromal lamellar organization in vivo. Ophthalmic Physiol. Opt..

[57] Lipari E., Sborgia A., Nubile M., Mastropasqua L., Alessio G. (2018). Polarimetric interferometry to objectively evaluate the optical properties of corneal stroma. J. Model. Ophthalmol..

[58] Sobczak M., Owczarek M., Woźniak W.A., Kurzynowski P. (2021). In vivo measurements of corneal birefringence properties using the one-way reflective Mueller polarimetry. Opt. Express.

[59] Knighton R.W., Huang X.R., Cavuoto L.A. (2008). Corneal birefringence mapped by scanning laser polarimetry. Opt. Express.

[60] Pircher M., Hitzenberger C.K., Schmidt-Erfurth U. (2011). Polarization sensitive optical coherence tomography in the human eye. Prog. Retin. Eye Res..

[61] Patil R.M., Shetty R.M., Narasimhan R.M., Patel Y.M., Khamar P., Pircher M., Hitzenberger C.K., Nuijts R.M., Roy A.S. (2022). Mapping of corneal birefringence in thin and asymmetric keratoconus corneas with ultrahigh-resolution polarization-sensitive OCT. J. Cataract Refract. Surg..

[62] Misson G.P. (2012). Birefringent Properties of the Human Cornea In Vivo: Towards a New Model of Corneal Structure. Ph.D. Thesis.

[63] Mastropasqua R., Nubile M., Salgari N., Lanzini M., Calienno R., Mattei P.A., Sborgia A., Agnifili L. (2017). Interference figures of polarimetric interferometry analysis of the human corneal stroma. PLoS ONE.

[64] Sobczak M., Asejczyk M. (2021). Birefringent properties of the cornea measured by a Mueller type polarimeter in healthy adults and children. Biomed. Opt. Express.

[65] Pierscionek B.K., Weale R.A. (1997). Is there a link between corneal structure and the ‘corneal cross’?. Eye.

[66] Pierscionek B.K., Weale R.A. (1998). Investigation of the polarization optics of the living human cornea and lens with purkinje images. Appl. Opt..

[67] Hecht E., Hecht E. (1987). Polarisation. Optics.

[68] Stoiber R.E., Morse S.A., Stoiber R.E., Morse S.A. (1994). Biaxial crystal optics. Crystal Identification with the Polarizing Microscope.

[69] Misson G.P. (2010). The theory and implications of the biaxial model of corneal birefringence. Ophthalmic Physiol. Opt..

[70] Jaronski J.W., Kasprzak H.T. (2003). Linear birefringence measurements of the in vitro human cornea. Ophthalmic Physiol. Opt..

[71] Van Blokland G.J., Verhelst S.C. (1987). Corneal polarization in the living human eye explained with a biaxial model. J. Opt. Soc. Am. A.

[72] Donohue D.J., Stoyanov B.J., McCally R.L., Farrell R.A. (1996). A numerical test of the normal incidence uniaxial model of corneal birefringence. Cornea.

[73] Fanjul-Vélez M., Pircher M., Baumann B., Götzinger E., Arce-Diego J.L., Hitzenberger C.K. (2009). Modelling human corneal polarization properties and comparison with PS-OCT measurements. Ophthalmic Technologies XIX.

[74] Sobczak M., Jóźwik A., Kurzynowski P. (2024). An integrated model of the human cornea as a linear biaxial birefringent medium. Sci. Rep..

[75] Götzinger E., Pircher M., Sticker M., Fercher A.F., Hitzenberger C.K. (2004). Measurement and imaging of birefringent properties of the human cornea with phase-resolved, polarization-sensitive optical coherence tomography. J. Biomed. Opt..

[76] Hitzenberger C.K., Götzinger E., Pircher M. (2006). Birefringence properties of the human cornea measured with polarization sensitive optical coherence tomography. Bull. Soc. Belg. Belge. Ophtalmol..

[77] Greenfield D.S., Knighton R.W. (2001). Stability of corneal polarization axis measurements for scanning laser polarimetry. Ophthalmology.

[78] Irsch K., Shah A.A. (2012). Birefringence of the central cornea in children assessed with scanning laser polarimetry. J Biomed. Opt..

[79] Shurcliff W.A., Shurcliff W.A. (1966). Overview of Mueller and Jones calculi. Polarized Light.

[80] Lvovsky A.I., Hoffman C., Driggers R. (2013). Fresnel Equations. Encyclopedia of Optical Engineering.

[81] Fischer F.P. (1928). Uber die darstellung der hornhautoberflache und ihrer veranderungen im reflexbild. Arch. Augenheilkunde.

[82] Fischer F.P. (1933). Bermerkungen zur.hartingers artukel uber Zeis­sischen cornealreflectographen und seine hornhautbilder. Ophthalmol. Optik..

[83] Tiffany J.M. (1986). Refractive index of meibomian and other lipids. Curr. Eye Res..

[84] Cope W.T., Wolbarsht M.L., Yamanashi B.S. (1978). The corneal polarization cross. J. Opt. Soc. Am..

[85] Stanworth A., Naylor E.J. (1950). The polarization optics of the isolated cornea. Br. J. Ophthalmol..

[86] Bueno J.M., Jaronski J. (2001). Spatially resolved polarization properties for in vitro corneas. Ophthalmic Physiol. Opt..

[87] Atchison D.A., Smith G., Atchison D.A., Smith G. (2000). Passage of light into the eye. Optics of the Human Eye.

[88] Bueno J.M., Berrio E., Artal P. (2006). Corneal polarimetry after LASIK refractive surgery. J. Biomed. Opt..

[89] Bueno J.M., Vargas-Martín F. (2002). Measurements of the corneal birefringence with a liquid-crystal imaging polarizcope. Appl. Opt..

[90] Bueno J.M. (2000). Measurement of parameters of polarization in the living human eye using imaging polarimetry. Vis. Res..

[91] Sobczak M., Asejczyk M., Wilczyński M. (2023). The effect of pupil size on the measurement of corneal birefringence properties: Preliminary study. Sci. Rep..

[92] Deshmukh R., Mohan R.M., Madonadi Kuniyil V., Vaddavalli P.K. (2025). Porcine Collagen Implants in Advanced Keratoconus: Outcomes at 1 Year. J. Refract. Surg..

[93] Baker T.Y. (1943). Ray tracing through non-spherical surfaces. Proc. Phys. Soc..

